# Effects of Metoprolol on Periprocedural Myocardial Infarction After Percutaneous Coronary Intervention (Type 4a MI): An Inverse Probability of Treatment Weighting Analysis

**DOI:** 10.3389/fcvm.2021.746988

**Published:** 2021-11-23

**Authors:** Duanbin Li, Ya Li, Maoning Lin, Wenjuan Zhang, Guosheng Fu, Zhaoyang Chen, Chongying Jin, Wenbin Zhang

**Affiliations:** ^1^Department of Cardiology, Sir Run Run Shaw Hospital, College of Medicine, Zhejiang University, Hangzhou, China; ^2^Key Laboratory of Cardiovascular Intervention and Regenerative Medicine of Zhejiang Province, Hangzhou, China; ^3^Department of Information Technology, Sir Run Run Shaw Hospital, College of Medicine, Zhejiang University, Hangzhou, China; ^4^Department of Cardiology, Union Hospital, Fujian Medical University, Fuzhou, China

**Keywords:** coronary artery disease, percutaneous coronary intervention, periprocedural myocardial infarction, β-blocker, metoprolol

## Abstract

**Background:** Metoprolol is the most used cardiac selective β-blocker and has been recommended as a mainstay drug in the management of acute myocardial infarction (AMI). However, the evidence supporting this regimen in periprocedural myocardial infarction (PMI) is limited.

**Methods:** This study identified 860 individuals who suffered PMI following percutaneous coronary intervention (PCI) procedure and median followed up for 3.2 years. Subjects were dichotomized according to whether they received chronic oral sustained-release metoprolol succinate following PMI. After inverse probability of treatment weighting (IPTW) adjustment, logistic regression analysis, Kaplan-Meier curve, and Cox regression analysis were performed to estimate the effects of metoprolol on major adverse cardiovascular events (MACEs) which composed of cardiac death, myocardial infarction (MI), stroke, and revascularization. Moreover, an exploratory analysis was performed according to hypertension, cardiac troponin I (cTnI) elevation, and cardiac function. A double robust adjustment was used for sensitivity analysis.

**Results:** Among enrolled PMI subjects, 456 (53%) patients received metoprolol treatment and 404 (47%) patients received observation. After IPTW adjustment, receiving metoprolol was found to reduce the subsequent 3-year risk of MACEs by nearly 7.1% [15 vs. 22.1%, absolute risk difference (ARD) = 0.07, number needed to treat (NNT) = 14, relative risk (RR) = 0.682]. In IPTW-adjusted Cox regression analyses, receiving metoprolol was related to a reduced risk of MACEs (hazard ratio [HR] = 0.588, 95%CI [0.385–0.898], *P* = 0.014) and revascularization (HR = 0.538, 95%CI [0.326–0.89], *P* = 0.016). Additionally, IPTW-adjusted logistic regression analysis showed that receiving metoprolol reduced the risk of MI at the third year (odds ratio [OR] = 0.972, 95% CI [0.948–997], *P* = 0.029). Exploratory analysis showed that the protective effect of metoprolol was more pronounced in subgroups of hypertension and cTnI elevation ≥1,000%, and was remained in patients without cardiac dysfunction. The benefits above were consistent when double robust adjustments were performed.

**Conclusion:** In the real-world setting, receiving metoprolol treatment following PCI-related PMI has decreased the subsequent risk of MACEs, particularly the risk of recurrent MI and revascularization.

## Background

Coronary artery disease contributes significantly to cardiovascular disease being the leading cause of death around the world (1). Over the past decades, coronary revascularization by the percutaneous coronary intervention (PCI) has been an established therapeutic procedure of coronary artery disease (CAD) (2). However, a silent “killer” still exists. Approximately 3–6% of patients experienced a periprocedural myocardial infarction (PMI) following PCI procedure and up to one-third of patients suffered periprocedural myocardial injury (3, 4). According to the 4th Universal Definition of Myocardial Infarction (UDMI), myocardial infarction (MI) associated with PCI is categorized as type 4a MI, which is primarily determined by the elevation level of cardiac troponin I (cTnI) (5). Numerous studies have demonstrated that PMI is related to the subsequent increased risk of mortality and other adverse cardiovascular events (6). Indeed, even periprocedural myocardial injury has been shown to increase the all-cause mortality following PCI procedure (7). The mechanisms of PCI-related PMI involve acute side branch occlusion, distal embolization, and mechanical process resulting in vulnerable plaque rupture (8). However, the eligible treatment strategy for PCI-related PMI remains in debate. Treatment strategies of acute myocardial infarction (AMI) may benefit PCI-related PMI, but the evidence is limited.

As a competitive and reversible antagonist of beta-1-adrenergic receptors, metoprolol has been the most used β-blocker with over 50 million total prescriptions per year in the U.S. (9). In the Goteborg Metoprolol Trial, metoprolol therapy initiated on admission reduced 3-month mortality and exert a prophylactic effect against ventricular fibrillation in patients with AMI (10, 11). A subsequent study found that long-term administration of 100 mg twice daily of metoprolol reduced the risk of cardiac death and non-fatal reinfarction in patients surviving AMI (12). Besides, early intravenous metoprolol before reperfusion was shown to reduce infarct size and improve left ventricular ejection fraction (LVEF) after ST-segment elevation myocardial infarction (STEMI) (13). The cardiovascular protective effect of metoprolol is established, which is achieved by inhibiting the overactive adrenergic nervous system, reducing oxygen demand, increasing cardiac perfusion, and reducing ventricular remodeling (14). The current clinical guidelines of AMI recommend the β-blockers administration as early as possible and continue thereafter, regardless of STEMI or non-STEMI (15, 16).

Therefore, this real-world multicentric cohort study was conducted to estimate the effects of metoprolol on PMI associated with PCI (type 4a MI) and to optimize clinical decisions.

## Methods

### Study Population

**Figure 1:**
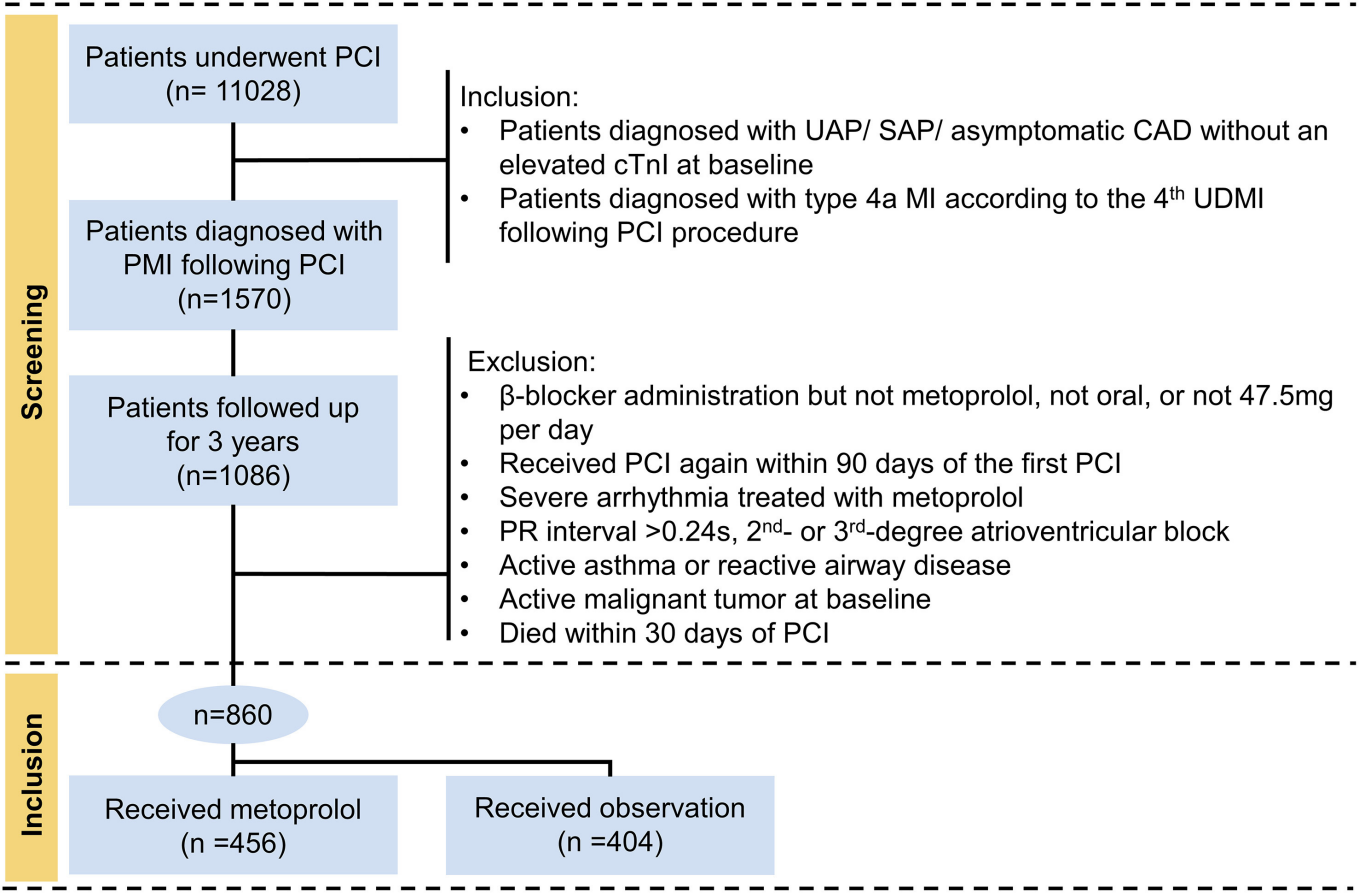
Flowchart describing the selection of subjects. ULN, the upper limit of normal; cTnI, cardiac troponin I; PCI, percutaneous coronary intervention; PMI, periprocedural myocardial infarction; UAP, unstable angina pectoris; SAP, stable angina pectoris; CAD, coronary artery disease.

This was a multicentric retrospective cohort study in the real-world setting. According to the 4th UDMI (5), a total of 1,570 patients diagnosed with PMI following PCI (type 4a MI) were eligible for this study from January 2014 to September 2018. Inclusion to the study required to meet the following criteria: (1) patients diagnosed with unstable angina pectoris (UAP)/stable angina pectoris (SAP)/asymptomatic CAD without an elevated cTnI at baseline; (2) patients who suffered from PCI-related PMI were followed up for 3 years. In contrast, the following patients were excluded: (1) β-blocker administration but not metoprolol, not oral, or not 47.5 mg daily; (2) received PCI again within 90 days of the first PCI; (3) treated with metoprolol due to severe arrhythmia; (4) prolonged PR intervals (>0.24 s), second- or third-degree atrioventricular blocks; (5) active asthma or reactive airway disease; (6) active malignant tumor at baseline; (7) died within 30 days of PCI. The final study population thus included 860 individuals (Figure 1). Ethical approval was granted by the Ethics Committee of Sir Run Run Hospital, College of Medicine, Zhejiang University (20201217-36).

### Percutaneous Coronary Intervention

The decision to perform treatments was made by the physician and the patient in consultation, and the procedure and the placement location of stents were entirely up to the currently recommended guidelines (17). Patients were treated with the optimal strategy of medications, including dual antiplatelet drugs, anticoagulants, and lipid-lowering therapy (18).

### Periprocedural Myocardial Infarction

The peak value of cTnI was determined by repeated laboratory examination within 48 h following the PCI procedure and was used to diagnose PMI. The upper limit of normal (ULN) of cTnI was determined at 0.011 ng/ml. The criteria of cTnI in PMI was as a post-procedural cTnI ≥5 × ULN with a normal cTnI at baseline or ≥20% cTnI elevation for patients with an elevated cTnI at baseline. Additionally, ischemic symptoms, ECG changes, angiography, or imaging abnormal should be verified according to the 4th UDMI in 2018 (5).

### Definition of Exposure

Among the enrolled population, subjects were dichotomized according to treatment strategy. Those who initiated metoprolol treatment at the acute phase of PMI and continued thereafter were categorized into the metoprolol treatment group. Alternatively, the observation group consisted of patients who did not receive metoprolol treatment. Medication data were extracted from the electronic medical record system and verified by telephone interview. Standard metoprolol exposure was defined as oral sustained-release metoprolol succinate 47.5 mg per day.

### Other Covariates

The study abstracted patient-level variables, including demographic features, laboratory data, PCI-related data, medications in hospitalization, and medications after discharge. Data of PCI treatment was also abstracted, including chronic coronary total occlusions (CTO), lesion location, number of stents, and direct PCI. International Classification of Diseases, 9th Revision, Clinical Modification (ICD-9-CM) was employed to define diabetes mellitus (DM) (ICD-9-CM 250) and hypertension (ICD-9-CM 362.11, 401–405, and 437.2). Normal NT-proBNP was defined as follows: <50 years old, <450 ng/L; 50–75 years old, <900 ng/L; and >75 years old, <1,800 ng/L.

### Endpoints

After discharge, telephone interviews were performed at every 6-month intervals by trained interviewers. The primary analytical endpoint of the study was major adverse cardiovascular events (MACEs), which was consisted of cardiac death, MI, stroke, and revascularization.

### Statistical Analyses

First, continuous variables were shown as the mean ± *SD* and were compared using Mann-Whitney *U*-tests. Categorical variables were represented as counts (proportions) and were compared using the Chi-square test or Fisher's exact test (if the expected cell value was <5). Missing data were replaced by single imputation with the median value of the cohort. Among these, NT-proBNP had the largest proportion of missing values (4.65%), followed by ejection fraction (EF) (3.49%), C-reactive protein (CRP) (2.91%), direct PCI (2.09%), uric acid (1.63%), the peak value of creatine kinase MB (CK-MB) (1.16%), lipoprotein (a) (1.16%), and very-low-density lipoprotein (VLDL) (1.05%).

Second, to minimize the selection bias, inverse probability of treatment weighting (IPTW) was applied to balance baseline characteristics between cohorts (19). In the IPTW approach, the propensity score (PS) was calculated by a logistic regression model, which predicted the probability of each individual receiving metoprolol. Then each individual was weighted according to PS. The model of PS component variables and their respective weights were shown in Supplementary Table S1. The balance of covariates was assessed by standardized mean difference (SMD) with imbalance defined as SMD >0.1 (20). Besides, IPTW-adjusted PS distribution was depicted in each cohort by kernel density plot.

Third, absolute risk difference (ARD), relative risk difference (RRD), number needed to treat (NNT), and relative risk (RR) were estimated in the IPTW-adjusted population. The NNT was the reciprocal of the ARD and indicated how many persons on average need to be exposed to metoprolol treatment to cause benefit in one person who would not otherwise have been benefited (21).

Fourth, IPTW-adjusted logistic regression analyses were conducted at first, second, and third year to assess the effects of metoprolol in population without censored data. After IPTW adjustment, Kaplan-Meier analysis and Cox regression analysis were further performed.

Fifth, an exploratory analysis was conducted to determine the IPTW-adjusted odds ratio (OR) and hazard ratio (HR) of metoprolol treatment according to hypertension (yes or no), the elevation of cTnI (<1,000% or ≥1,000%), and cardiac function (EF ≤ 50% or EF ≥50% with normal NT-proBNP) after rebalancing every covariate in subgroups by the approach depicted above.

Sixth, a sensitivity analysis was conducted applying a double robust approach (IPTW with multivariate regression adjustment) (22). Covariates with SMD >0.05 after IPTW adjustment were further adjusted in multivariate regression models.

Statistical analysis was conducted by SPSS software version 18 (SPSS Inc., Chicago, IL, USA) and R version 3.5.1 (The R Foundation for Statistical Computing, Vienna, Austria). Two-sided statistical significance was defined as *P* < 0.05.

## Results

### Patient Characteristics

A total of 860 patients were identified as PMI by the definition of the 4th UDMI and followed up for over 3 years. Among them, 456 (53%) received metoprolol and 404 (47%) received observation, respectively (Table 1). Patients who received metoprolol were younger (67.4 ± 10.7 vs. 70.1 ± 10.3 years, *P* < 0.001), had higher levels of lipoprotein (a) (27.1 ± 27.4 vs. 22.6 ± 22.2 mg/dl, *P* = 0.011), higher prevalence of CTO (13.6 vs. 8.2%, *P* = 0.015), were more likely to receive angiotensin-converting enzyme inhibitor (ACEI) treatment (42.3 vs. 33.7%, *P* = 0.011), and were less likely to receive calcium channel blocker (CCB) treatment (25.0 vs. 35.1%, *P* = 0.002). However, there was no significant difference between metoprolol and observation groups in the peak value of cTnI (1.87 ± 1.86 vs. 1.91 ± 1.94 mg/L, *P* = 0.776), the peak value of CK-MB (28.1 ± 20.9 vs. 25.8 ± 20.5 U/L, *P* = 0.116), and angiotensin receptor blocker (ARB) treatment (37.9 vs. 41.6%, *P* = 0.307).

**Table 1:** Unweighted baseline characteristics.

**Patient characteristics**	**Overall**	**Observation**	**Metoprolol**	***P*-value**
	***n* = 860**	***n* = 404**	***n* = 456**	
**Demographic features**				
Age, years	68.69 ± 10.59	70.13 ± 10.30	67.42 ± 10.70	<0.001^*^
Male, *n* (%)	604 (70.7)	290 (72.1)	314 (69.5)	0.435
Diabetes, *n* (%)	227 (26.4)	101 (25.0)	126 (27.6)	0.426
Hypertension, *n* (%)	621 (72.2)	299 (74.0)	322 (70.6)	0.301
Current smoker, *n* (%)	210 (24.4)	107 (26.5)	103 (22.6)	0.212
Current drinker, *n* (%)	138 (16.0)	80 (19.8)	58 (12.7)	0.006^*^
BMI, kg/m^2^	24.49 ± 3.09	24.40 ± 3.16	24.56 ± 3.03	0.435
Prior MI, *n* (%)	89 (10.3)	37 (9.2)	52 (11.4)	0.334
Prior PCI, *n* (%)	257 (29.9)	116 (28.7)	141 (30.9)	0.528
Ejection fraction, %	63.29 ± 11.58	64.74 ± 11.45	62.21 ± 11.57	0.006^*^
Heart rate on admission, beats/min	74.2 ± 12.3	72.6 ± 12.1	75.6 ± 12.3	0.001^*^
Systolic blood pressure, mmHg	133.68 ± 20.54	134.36 ± 20.94	133.08 ± 20.18	0.364
Diastolic blood pressure, mmHg	73.71 ± 12.11	73.60 ± 11.90	73.80 ± 12.31	0.803
Length of hospitalization, days	4.51 ± 4.25	4.58 ± 3.16	4.44 ± 5.03	0.634
**Clinical presentation**				0.608
Unstable angina pectoris	472 (54.9)	227 (56.2)	245 (53.7)	
Stable angina pectoris	317 (36.9)	142 (35.1)	175 (38.4)	
Asymptomatic CAD	71 (8.2)	35 (8.7)	36 (7.9)	
**Laboratory data**				
Peak value of CK-MB, U/L	26.97 ± 20.73	25.76 ± 20.45	28.05 ± 20.94	0.116
Peak value of cTnI, mg/L	1.89 ± 1.90	1.91 ± 1.94	1.87 ± 1.86	0.776
NT-proBNP, pg/mL	1310.31 ± 3010.94	1358.53 ± 3000.53	1270.56 ± 3025.05	0.759
Total cholesterol, mg/dL	3.87 ± 1.04	3.91 ± 1.05	3.83 ± 1.03	0.235
Low density lipoprotein, mg/dL	2.04 ± 0.84	2.07 ± 0.83	2.01 ± 0.84	0.311
Very low density lipoprotein, mg/dL	0.94 ± 1.41	0.93 ± 1.34	0.95 ± 1.47	0.889
High density lipoprotein, mg/dL	1.00 ± 0.28	1.00 ± 0.27	1.01 ± 0.28	0.826
Triglyceride, mg/dL	1.60 ± 1.01	1.61 ± 1.14	1.58 ± 0.89	0.660
Lipoprotein (a), mg/dL	25.00 ± 25.14	22.62 ± 22.17	27.11 ± 27.35	0.011^*^
Uric acid, μmol/L	368.73 ± 101.80	370.64 ± 101.54	367.00 ± 102.12	0.612
Fasting blood glucose, mmol/L	6.72 ± 2.61	6.67 ± 2.50	6.78 ± 2.71	0.534
Total bilirubin, μmol/L	12.70 ± 6.56	12.47 ± 5.56	12.91 ± 7.32	0.324
C-reactive protein, mg/L	6.18 ± 15.40	5.81 ± 15.32	6.48 ± 15.47	0.564
Platelet, × 10^9^/L	171.05 ± 54.54	169.49 ± 52.90	172.42 ± 55.97	0.431
Hemoglobin, g/dL	12.70 ± 1.85	12.74 ± 1.93	12.66 ± 1.78	0.533
White blood cell, × 10^9^/L	6.78 ± 2.24	6.76 ± 2.17	6.79 ± 2.30	0.824
**PCI data**				
CTO, *n* (%)	95 (11.0)	33 (8.2)	62 (13.6)	0.015^*^
Lesion location, *n* (%)				
LM	84 (9.8)	42 (10.4)	42 (9.2)	0.639
LCX	187 (21.7)	81 (20.0)	106 (23.2)	0.293
LAD	479 (55.7)	226 (55.9)	253 (55.5)	0.947
RCA	206 (24.0)	98 (24.3)	108 (23.7)	0.907
No of stents ≥2, *n* (%)	490 (57.0)	219 (54.2)	271 (59.4)	0.140
Direct PCI, *n* (%)	76 (10.3)	34 (10.2)	42 (10.5)	0.993
**Medication**, ***n*** **(%)**				
DAPT	811 (94.3)	381 (94.3)	430 (94.3)	1.000
Statin	853 (99.2)	400 (99.0)	453 (99.3)	0.872
Trimetazidine	289 (33.6)	136 (33.7)	153 (33.6)	1.000
ACEI	329 (38.3)	136 (33.7)	193 (42.3)	0.011^*^
ARB	341 (39.7)	168 (41.6)	173 (37.9)	0.307
CCB	256 (29.8)	142 (35.1)	114 (25.0)	0.002^*^

*PMI, periprocedural myocardial infarction; BMI, body mass index; MI, myocardial infarction; PCI, percutaneous coronary intervention; NTproBNP, N-terminal pro-brain natriuretic peptide; CABG, coronary artery bypass grafting; CK-MB, creatine kinase MB; cTnI, cardiac troponin I; CTO, chronic coronary total occlusions; LM, left main coronary artery; LCX, left circumflex coronary artery; LAD, left anterior descending coronary artery; RCA, right coronary artery; DAPT, dual antiplatelet therapy; ACEI, angiotensin-converting enzyme inhibitor; ARB, angiotensin receptor blocker; CCB, calcium channel blocker*.

* *P < 0.05*.

### Patient Characteristics After IPTW Adjustment

**Figure 2:**
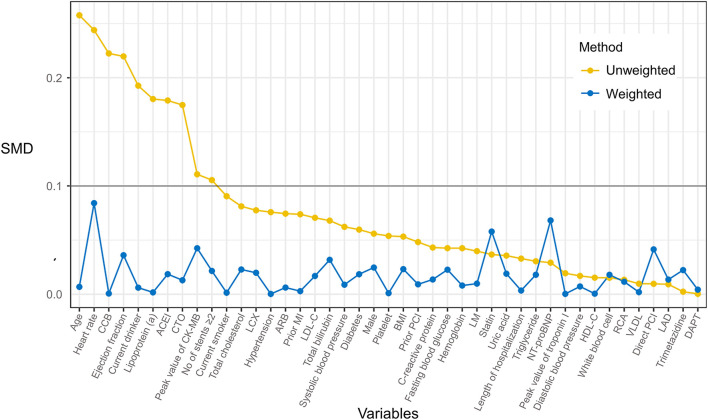
Effect of inverse probability of treatment weighting (IPTW) on baseline characteristics distribution of PMI patients who received metoprolol vs. observation. The imbalance between treatment groups was defined as a standardized mean difference (SMD) >0.1.

After IPTW adjustment, there was no significant difference between cohorts in demographic features, laboratory data, PCI data, and medications. Baseline characteristics after IPTW adjustment were listed in Table 2. Supplementary Table S1 showed the multivariable logistic regression model that predicted the probability of receiving metoprolol. By using the IPTW method, the SMD of each covariate was below 0.1, indicating that cohorts were comparable thereafter (Figure 2). Besides, distributions of PS between cohorts reached a sufficient balance after IPTW adjustment (Supplementary Figure S1).

**Table 2:** Baseline characteristics of IPTW-adjusted population.

**Patient characteristics**	**Overall**	**Observation**	**Metoprolol**	***P*-value**
	***n* = 860**	***n* = 404**	***n* = 456**	
**Patient characteristics**				
Age, years	67.95 ± 10.25	67.95 ± 10.19	67.94 ± 10.32	0.993
Male, *n* (%)	806.7 (69.0)	396.1 (68.5)	410.6 (69.5)	0.805
Diabetes, *n* (%)	290.5 (24.9)	142.2 (24.6)	148.3 (25.1)	0.891
Hypertension, *n* (%)	844.8 (72.3)	416.9 (72.1)	427.9 (72.5)	0.926
Current smoker, *n* (%)	266.1 (22.8)	131.3 (22.7)	134.8 (22.8)	0.973
Current drinker, *n* (%)	184.0 (15.7)	90.0 (15.6)	94.0 (15.9)	0.914
BMI, kg/m^2^	24.46 ± 3.04	24.42 ± 3.12	24.50 ± 2.97	0.750
Prior MI, *n* (%)	123.5 (10.6)	62.2 (10.7)	61.3 (10.4)	0.889
Prior PCI, *n* (%)	326.2 (27.9)	162.9 (28.2)	163.4 (27.7)	0.901
Ejection fraction, %	63.56 ± 11.39	63.78 ± 11.58	63.33 ± 11.23	0.656
Heart rate on admission, beats/min	75.2 ± 12.0	74.4 ± 11.9	75.8 ± 12.1	0.257
Systolic blood pressure, mmHg	134.17 ± 20.92	134.18 ± 20.87	134.15 ± 21.01	0.986
Diastolic blood pressure, mmHg	74.19 ± 12.51	74.13 ± 12.35	74.25 ± 12.68	0.910
Length of hospitalization, days	4.46 ± 4.56	4.45 ± 3.34	4.47 ± 5.50	0.970
**Laboratory data**				
Peak value of CK-MB, U/L	30.17 ± 21.46	30.74 ± 21.53	29.61 ± 21.41	0.572
Peak value of troponin I, mg/L	1.79 ± 1.75	1.80 ± 1.76	1.79 ± 1.74	0.991
NT-proBNP, pg/mL	1171.90 ± 2488.13	1254.29 ± 2482.29	1091.57 ± 2495.51	0.537
Total cholesterol, mg/dL	3.93 ± 1.08	3.95 ± 1.10	3.92 ± 1.06	0.739
Low density lipoprotein, mg/dL	2.11 ± 0.84	2.11 ± 0.85	2.10 ± 0.84	0.808
Very low density lipoprotein, mg/dL	0.95 ± 1.43	0.94 ± 1.39	0.95 ± 1.47	0.943
High density lipoprotein, mg/dL	1.00 ± 0.28	0.99 ± 0.28	1.00 ± 0.27	0.971
Triglyceride, mg/dL	1.55 ± 0.87	1.55 ± 0.89	1.56 ± 0.85	0.807
Lipoprotein (a), mg/dL	26.52 ± 25.71	26.55 ± 23.80	26.49 ± 27.50	0.980
Uric acid, μmol/L	370.56 ± 104.79	369.47 ± 103.75	371.63 ± 105.97	0.818
Fasting blood glucose, mmol/L	6.64 ± 2.62	6.62 ± 2.61	6.66 ± 2.64	0.868
Total bilirubin, μmol/L	12.74 ± 6.58	12.62 ± 5.99	12.86 ± 7.12	0.665
C-reactive protein, mg/L	6.14 ± 15.44	6.02 ± 16.14	6.27 ± 14.74	0.856
Platelet, × 10^9^/L	171.17 ± 54.74	170.80 ± 53.44	171.53 ± 56.08	0.879
Hemoglobin, g/dL	12.73 ± 1.84	12.73 ± 1.91	12.74 ± 1.77	0.947
White blood cell, × 10^9^/L	6.68 ± 2.10	6.67 ± 1.98	6.70 ± 2.21	0.896
**PCI data**				
CTO, *n* (%)	139.4 (11.9)	67.4 (11.7)	71.9 (12.2)	0.864
Lesion location, *n* (%)				
LM	111.0 (9.5)	55.9 (9.7)	55.1 (9.3)	0.907
LCX	236.8 (20.3)	116.3 (20.1)	120.5 (20.4)	0.939
LAD	657.6 (56.3)	326.3 (56.4)	331.3 (56.1)	0.944
RCA	304.6 (26.1)	152.3 (26.3)	152.3 (25.8)	0.889
No of stents ≥2, *n* (%)	730.5 (62.5)	370.5 (64.1)	360.0 (61.0)	0.470
Direct PCI, *n* (%)	105.5 (9.7)	55.5 (10.2)	50.0 (9.2)	0.725
**Medication**, ***n*** **(%)**				
DAPT	1102.5 (94.3)	547.6 (94.7)	555.0 (94.0)	0.720
Statin	1167.8 (99.9)	578.4 (100.0)	589.4 (99.8)	0.324
Trimetazidine	423.1 (36.2)	205.7 (35.6)	217.4 (36.8)	0.768
ACEI	438.2 (37.5)	215.1 (37.2)	223.1 (37.8)	0.893
ARB	455.9 (39.0)	216.7 (37.5)	239.3 (40.5)	0.478
CCB	333.8 (28.6)	164.9 (28.5)	169.0 (28.6)	0.979

*reference to Table 1*.

### Metoprolol vs. Observation

A total of 165 (19.2%) MACEs occurred during the 3-year follow-up period. After IPTW adjustment, individuals who received metoprolol reduced the subsequent risk of MACEs by nearly 7.1% (15 vs. 22.1%, ARD = 0.07, RRD = 0.318, NNT = 14, RR = 0.682), recurrent MI by nearly 2.6% (0.9 vs. 3.7%, ARD = 0.028, RRD = 0.766, NNT = 35, RR = 0.234), and revascularization by nearly 5.5% (10.4 vs. 15.9%, ARD = 0.055, RRD = 0.347, NNT = 18, RR = 0.653) (Table 3).

**Table 3:** Treatment effect of metoprolol vs. observation during the 3 years following the PCI-related PMI in IPTW-adjusted population.

	**Incidence rate % (events/patients)**		**ARD**	**RRD**	**NNT**	**RR**
	**Metoprolol**	**Observation**				
MACEs	15.0% (29.1/193.4)	22.1% (26.2/118.6)	0.070	0.318	14	0.682
Cardiac death	3.4% (6.7/193.4)	3.5% (4.1/118.6)	0.0003	0.009	3325	0.991
MI	0.9% (1.7/193.4)	3.7% (4.4/118.6)	0.028	0.766	35	0.234
Stroke	0.6% (1.2/193.4)	NA	NA	NA	NA	NA
Revascularization	10.4% (20.1/193.4)	15.9% (18.9/118.6)	0.055	0.347	18	0.653

*ARD, absolute risk difference; RRD, relative risk difference; NNT, number needed to treat; RR, relative risk; MACEs, major adverse cardiovascular events; NA, not available; others, reference to Table 1*.

IPTW-adjusted logistic regression (Table 4) showed that receipt of metoprolol significantly reduced the risk of MACEs at the second year (OR = 0.904, 95%CI [0.855–0.955], *P* < 0.001) and at the third year (OR = 0.925, 95%CI [0.868–0.986], *P* = 0.017). Specifically, administration of metoprolol significantly reduced the risk of revascularization at 1st year (OR = 0.966, 95%CI [0.937–0.995], *P* = 0.025), at the second year (OR = 0.931, 95%CI [0.887–0.977], *P* = 0.004), and at the third year (OR = 0.938, 95%CI [0.888–0.991], *P* = 0.022). Additionally, metoprolol treatment reduced the risk of recurrent MI at the third year following PMI (OR = 0.972, 95%CI [0.948–0.997], *P* = 0.029).

**Table 4:** Logistic regression analysis of metoprolol treatment on MACEs and its components at 1, 2, and 3 years following PCI-related PMI in IPTW-adjusted population.

	**3 years**			**2 years**			**1 year**		
	**Events/patients (%)**	**OR [95% CI]**	***P*-value**	**Events/patients (%)**	**OR [95% CI]**	***P*-value**	**Events/patients (%)**	**OR [95% CI]**	***P*-value**
MACEs	165/860 (19.2)	0.925 [0.868–0.986]	0.017^*^	123/860 (14.3)	0.904 [0.855–0.955]	<0.001^*^	46/860 (5.3)	0.970 [0.940–1.001]	0.059
Cardiac death	29/860 (3.4)	1.003 [0.973–1.034]	0.854	16/860 (1.9)	0.989 [0.968–1.011]	0.335	3/860 (0.3)	1.006 [0.997–1.015]	0.180
MI	12/860 (1.4)	0.972 [0.948–0.997]	0.029^*^	7/860 (0.8)	0.980 [0.959–1.002]	0.072	3/860 (0.3)	0.998 [0.989–1.007]	0.696
Stroke	3/860 (0.3)	1.006 [0.997–1.015]	0.175	2/860 (0.2)	1.002 [0.997–1.008]	0.395	0/860 (0)	NA	
Revascularization	125/860 (14.5)	0.938 [0.888–0.991]	0.022^*^	99/860 (11.5)	0.931 [0.887–0.977]	0.004^*^	40/860 (4.7)	0.966 [0.937–0.995]	0.025^*^

*MACEs, major adverse cardiovascular events; NA, not available; other, reference to Table 1*.

* *P < 0.05*.

**Figure 3:**
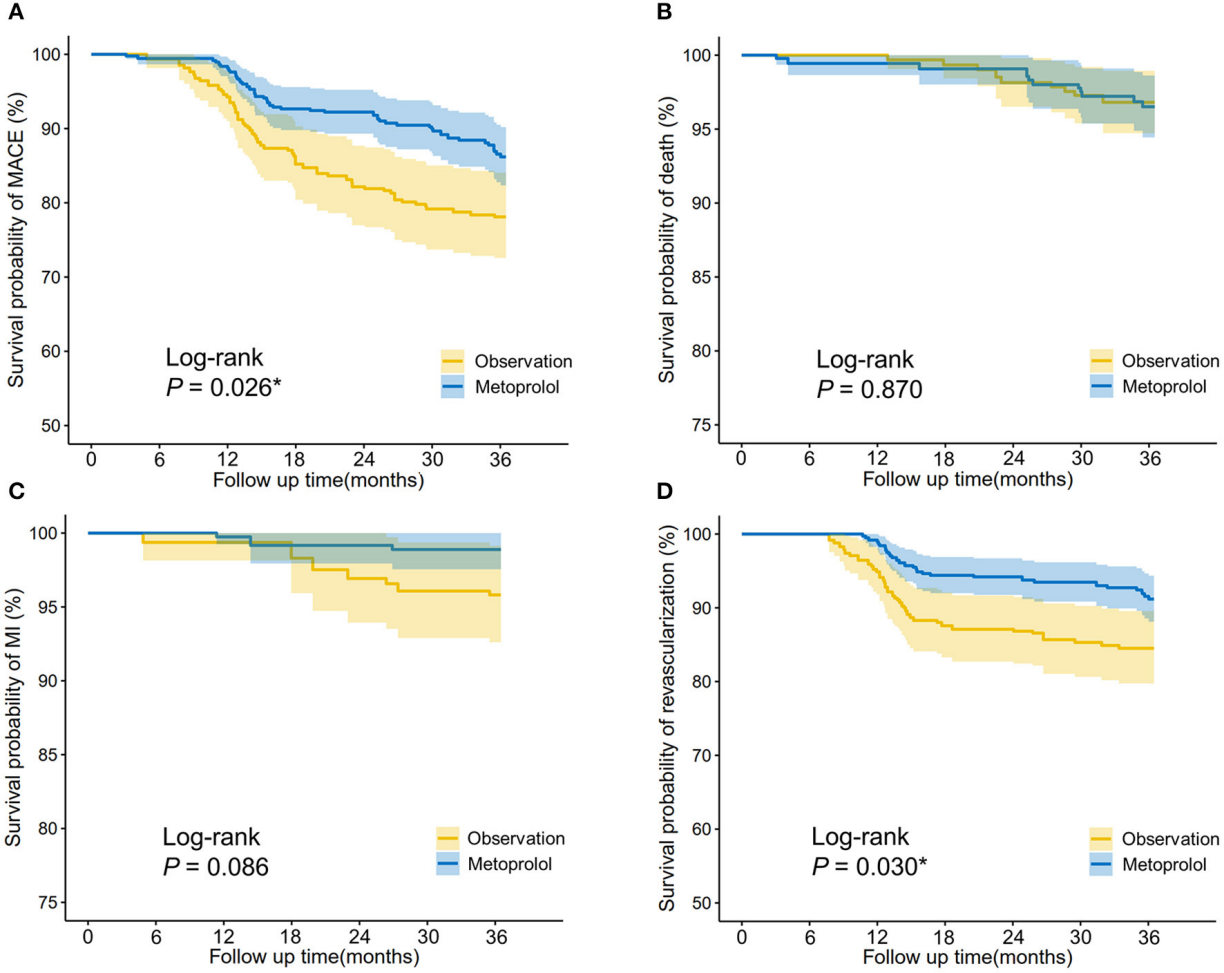
IPTW-adjusted Kaplan-Meier analysis of **(A)** major adverse cardiovascular events (MACEs), **(B)** cardiac death, **(C)** myocardial infarction, and **(D)** revascularization in patients who received metoprolol vs. observation after PCI-related PMI. The survival curves were indicated by solid lines and 95% CIs by shaded areas. Log-rank *P*-value was shown. *P < 0.05.

For patients who suffered PMI, IPTW-adjusted Kaplan-Meier curves (Figure 3) showed that the metoprolol group achieved a higher survival probability of MACEs (Log-rank *P* = 0.026) and revascularization (Log-rank *P* = 0.03) vs. the observation group. IPTW-adjusted Cox regression analyses indicated that receiving metoprolol treatment reduced the 3-year risk of MACEs (HR = 0.588, 95%CI [0.385–0.898], *P* = 0.014) and revascularization (HR = 0.538, 95%CI [0.326–0.89], *P* = 0.016; Table 5).

**Table 5:** Cox regression analysis of metoprolol treatment on MACEs and its components in unadjusted, IPTW adjusted, and double robust adjusted population.

	**Unadjusted**		**IPTW adjusted**		**Double robust adjusted**	
	**HR [95% CI]**	***P*-value**	**HR [95% CI]**	***P*-value**	**HR [95% CI]**	***P*-value**
MACEs	0.765 [0.560–1.044]	0.091	0.588 [0.385–0.898]	0.014^*^	0.651 [0.453–0.959]	0.025^*^
Cardiac death	0.825 [0.398–1.708]	0.604	1.201 [0.502–2.875]	0.681	1.057 [0.416–2.437]	0.910
MI	0.333 [0.088–1.253]	0.104	0.306 [0.076–1.233]	0.072	0.281 [0.057–1.124]	0.095
Stroke	NA		NA		NA	
Revascularization	0.753 [0.526–1.077]	0.120	0.538 [0.326–0.890]	0.016^*^	0.608 [0.393–0.968]	0.030^*^

*The double robust method additionally adjusted covariates with SMD ≥0.05 after IPTW adjustment, which was heart rate on admission, statin usage, and NT-proBNP. The coefficient of covariates was shown in Supplementary Table S3. MACEs, major adverse cardiovascular events; NA, not available; other, reference to Table 1*.

* *P < 0.05*.

### Subgroup Analyses

**Figure 4:**
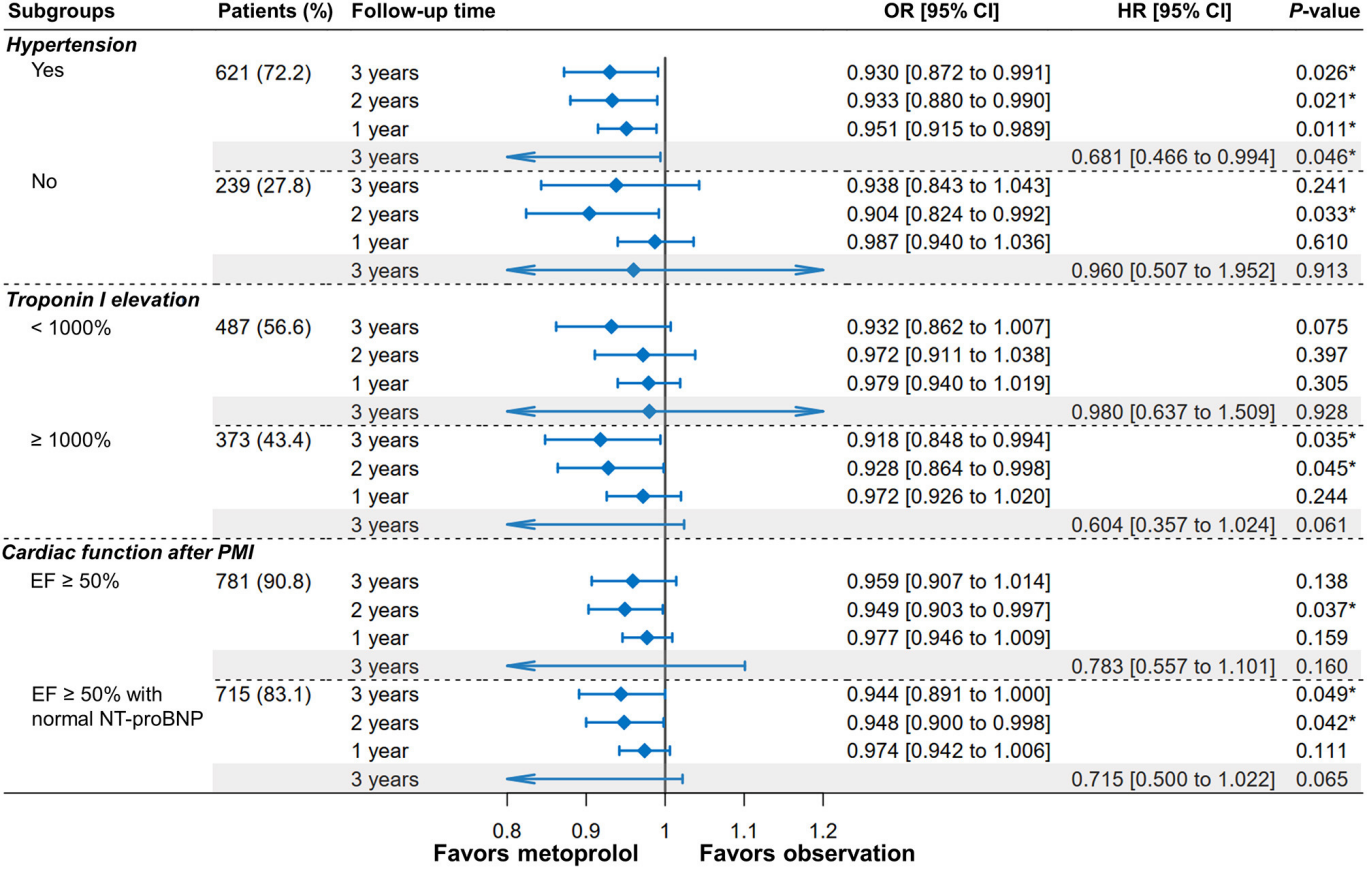
Forest plot of MACEs depicting the IPTW-adjusted odds ratios (ORs) and hazard ratios (HRs) of metoprolol administration vs. observation after PMI according to hypertension, troponin I elevation, and cardiac function. *P < 0.05.

Figure 4 shows the IPTW-adjusted ORs and HRs of receiving metoprolol vs. observation on MACEs according to hypertension, the elevation of cTnI, and cardiac function. Specifically, receipt of metoprolol was associated with a significantly reduced risk of MACEs in patients with hypertension (HR = 0.681, 95%CI [0.466–0.994], *P* = 0.046), which was independent of the use of other antihypertensive drugs (i.e., ACEI, ARB, and CCB). This protective effect has also been observed in non-hypertensive patients at the second year after PMI (OR = 0.904, 95%CI [0.824–0.992], *P* = 0.033). In patients with higher cTnI elevation, the protective effect was more pronounced and observed at the third year (OR = 0.918, 95%CI [0.848–0.994], *P* = 0.035) and the second year after PMI (OR = 0.928, 95%CI [0.864–0.998], *P* = 0.045). In patients without cardiac dysfunction (EF ≥50% with normal NT-proBNP), the benefit of metoprolol remained with the decreased risk of MACEs at the third year (OR = 0.944, 95% CI [0.891–1], *P* =0.049) and the second year (OR = 0.948, 95% CI [0.9–0.998], *P* = 0.042).

### Sensitivity Analyses

The association of metoprolol treatment with MACEs and its components remained in the sensitivity analysis using the double robust adjustment (IPTW with multivariable regression). Multivariable regression additionally adjusted covariates with SMD ≥0.05, which was heart rate on admission, statin usage, and NT-proBNP. The protective effect of metoprolol on MACEs (HR = 0.651, 95%CI [0.453–0.959], *P* = 0.025) and revascularization (HR = 0.608, 95%CI [0.393–0.968], *P* = 0.03) remained (Table 4). Consistently, metoprolol reduced the risk of MI at the third year (OR = 0.972, 95%CI [0.945–0.998], *P* = 0.037) after double robust adjustment (Supplementary Table S2).

## Discussion

This real-world multicentric study demonstrated the protective effects of metoprolol treatment for PCI-related PMI. Chronic receiving oral sustained-release metoprolol succinate reduces the subsequent 3-year risk of MACEs by nearly 7.1% than their counterparts who received observation, particularly the risk of recurrent MI and revascularization. The exploratory analysis showed that the protective effect of metoprolol was more pronounced in subgroups of hypertension and cTnI elevation ≥1,000%. Besides, the benefits of receiving metoprolol were observed to be consistent in patients without cardiac dysfunction.

Metoprolol is the most frequently used β-receptor blocker (9). In patients with AMI, the protective effect of metoprolol has been confirmed by numerous clinical studies, whether it is taken orally or intravenously (10–13). The clinical practice guidelines for AMI strongly recommend the use of β-blockers as soon as possible and continue to use thereafter, regardless of STEMI and non-STEMI (15, 16). The current study employed 47.5 mg daily oral sustained-release metoprolol succinate as exposure, which is <200 mg daily oral dose commonly used in previous RCTs (10–12). However, even a lower dose of metoprolol has been shown to reduce the risk of subsequent MACEs in the current study, which is consistent with the results of previous RCTs in AMI patients (10–13). In the current study, decreased risk of MACEs (nearly 7.1%) mainly came from the reduced risk of recurrent MI and revascularization, but not mortality. In contrast, for patients surviving AMI, chronic administration of β-blockers certainly leads to a reduction in subsequent mortality (23). This discrepancy may be because PMI is a minor myocardial infarction with a lower mortality rate, which masks the benefit of receiving β-blockers therapy.

The latest ESC clinical practice guidelines on AMI prefer to recommend (class IA) β-blockers administration when LVEF is ≤ 40%, regardless of STEMI or non-STEMI (15, 16). On the contrary, when LVEF is >40%, the evidence of benefits from β-blockers was limited. For this, several large clinical trials are underway to evaluate the effects of β-blockers on AMI patients without LVEF reduction (24). By propensity score matching, Choo et al. (25) confirmed a reduction in all-cause and cardiac mortality with β-blocker therapy at 3 years in AMI patients with EF ≥50%. Therefore, the current study additionally observed the effectiveness of receiving metoprolol treatment in subgroups of EF ≥50% and EF ≥50% with normal NT-proBNP. The results showed that the benefits of receiving metoprolol in reducing the risk of MACEs have remained and consistent with the main finding, which supports the chronic administration of metoprolol in PMI patients with preserved cardiac function.

Metoprolol is often used to lower blood pressure and has been shown to reduce subsequent mortality in the primary prevention of hypertensive patients (26). In the current study, the benefit of metoprolol for PMI patients seemed to be more pronounced in the subgroup of hypertension, which suggested that the benefit may partly come from the antihypertensive effect. However, the antihypertensive effect might not be the only mechanism. On the one hand, the benefits of metoprolol had also been observed in non-hypertensive patients despite relatively few subjects. On the other hand, the benefit of metoprolol was found in the overall population analysis after balancing the covariates between groups by IPTW adjustment (including hypertension, ACEI, ARB, and CCB). Therefore, the protective mechanism of metoprolol might partly come from the antihypertensive effect, but not all.

Periprocedural myocardial infarction is considered a minor MI. The previous study had shown that higher levels of cTnI were associated with a poorer prognosis (27). In this study, the protective effect of metoprolol appeared to be more pronounced in patients with a cTnI increase of ≥1,000%. This might be due to a greater cTnI elevation leading to a more significant prognostic difference, which in turn made the protective effect of metoprolol more prominent.

According to the fourth UDMI, MI was categorized into type 1 to type 5 considering the difference in pathology, clinical features, and prognosis, and therapeutic strategy (5). PCI-related PMI was termed type 4a MI, in which many complicated mechanisms intertwined including acute side branch occlusion, distal embolization, and mechanical process resulting in vulnerable plaque rupture (8, 28). For PCI-related PMI, the benefits from metoprolol may come from the following underlying mechanisms.

First, metoprolol may reduce the ischemia-reperfusion injury and infarct size of PMI, although it is a minor MI. In a porcine ischemia/reperfusion model, Ibanez et al. (29) proved that receiving metoprolol intravenously can reduce the size of MI. The subsequent METOCARD-CNIC trial demonstrated the protective effect of metoprolol administration before reperfusion in reducing infarct size and promoting prognosis in STEMI patients (13, 30). In the current study, patients had initiated to receive metoprolol at the acute phase of MI, which potentially reduce myocardial injury and thus improve the prognosis. Second, receiving metoprolol treatment in the acute phase of PMI may bring about more myocardial perfusion. In the acute phase of MI, β-blocker attenuates excessive sympathetic nervous system activity through a variety of mechanisms, including lowering heart rate to prolong diastolic periods, reducing cardiac contractility to reduce oxygen consumption, and dilating epicardial coronary arteries to increase coronary blood flow (14). Third, metoprolol may attenuate ventricular remodeling in patients surviving PMI and thus achieve long-term benefits (31). Unlike transmural necrosis in STEMI, subendocardial necrosis often appears in minor MI, which is prone to reverse remodeling in the setting of contractile reserve and revascularization (32). Through IPTW-adjustment, the current study balanced the potential inhibitor of cardiac remodeling (i.e., ACEI and ARB) between groups, which further supports the benefit of inhibiting remodeling from metoprolol.

In general, compared with AMI, PCI-related PMI can be deemed as a minor MI, which occurs mostly in patients with stable CAD who underwent index PCI. The current study found that PCI-related PMI patients can also benefit from the chronic administration of β-blockers, which was initially recommended for AMI patients by international guidelines.

### Limitation

First, this study was bound by inherent biases as a retrospective study. Second, 200 mg oral metoprolol was often used as the exposure dose in previous RCTs of AMI. However, due to the real-world setting, the current study identified 47.5 mg daily oral sustained-release metoprolol succinate as the exposure, thus its results may not be applicable to explain the effects of other doses of metoprolol on PMI. Third, revascularization was observed as a secondary endpoint, but target lesion revascularization or target vessel revascularization was not further analyzed. Fourth, the definition of PMI was adopted from the fourth UDMI based on cTnI, which cannot avoid the potential limitations of the definition of PMI.

## Conclusion

In the real-world setting, receiving metoprolol treatment following PCI-related PMI has decreased the subsequent risk of MACEs, particularly the risk of recurrent MI and revascularization.

## Data Availability Statement

The raw data supporting the conclusions of this article will be made available by the authors, without undue reservation.

## Ethics Statement

The studies involving human participants were reviewed and approved by Ethics Committee of Sir Run Run Shaw Hospital, College of Medicine, Zhejiang University (20201217-36). Written informed consent for participation was not required for this study in accordance with the national legislation and the institutional requirements.

## Author Contributions

WenbZ, CJ, and ZC conceived and designed the study. DL organized these data and drafted the manuscript with the help of ML. DL, YL, and WenjZ analyzed the data. YL drew the pictures. WenbZ, CJ, ZC, and GF detected any errors in the whole process. All authors have read and approved the manuscript for submission.

## Funding

This work was supported by grants from the National Natural Science Foundation of China (82070408 and 81800212), the Medical Health Science and Technology Project of Zhejiang Provincial Health Commission (2021RC014), the Traditional Chinese Medicine Science and Technology Project of Zhejiang Province (2021ZB172), and the Joint Funds for the innovation of science and Technology, Fujian province (2018Y9094).

## Conflict of Interest

The authors declare that the research was conducted in the absence of any commercial or financial relationships that could be construed as a potential conflict of interest.

## Publisher's Note

All claims expressed in this article are solely those of the authors and do not necessarily represent those of their affiliated organizations, or those of the publisher, the editors and the reviewers. Any product that may be evaluated in this article, or claim that may be made by its manufacturer, is not guaranteed or endorsed by the publisher.

## Abbreviations

CAD: coronary artery disease
PCI: percutaneous coronary intervention
PMI: periprocedural myocardial infarction
UDMI: universal definition of myocardial infarction
MI: myocardial infarction
AMI: acute myocardial infarction
cTnI: cardiac troponin I
β-blocker: β-adrenoceptor antagonist
LVEF: left ventricular ejection fraction
STEMI: ST-segment elevation myocardial infarction
ULN: the upper limit of normal
CTO: chronic total occlusion
ICD-9-CM: International Classification of Diseases, 9th Revision Clinical Modification
DM: diabetes mellitus
BMI: body mass index
NT-proBNP: N-terminal pro-brain natriuretic peptide
MACEs: major adverse cardiovascular events
EF: ejection fraction
CRP: C-reactive protein
CK-MB: creatine kinase MB
VLDL: very low-density lipoprotein
IPTW: inverse probability of treatment weighting
PS: propensity score
SMD: standardized mean difference
ARD: absolute risk difference
RRD: relative risk difference
NNT: number needed to treat
RR: relative risk
HR: hazard ratio
OR: odds ratio
ACEI: angiotensin-converting enzyme inhibitor
ARB: angiotensin receptor blocker
RCT: randomized controlled trial.
