# Supplementary material for: Effects of Metoprolol on Periprocedural Myocardial Infarction After Percutaneous Coronary Intervention (Type 4a MI): An Inverse Probability of Treatment Weighting Analysis
Source: Front Cardiovasc Med. 2021 Nov 23;8:746988. doi: 10.3389/fcvm.2021.746988 (PMC8650586; doi:10.3389/fcvm.2021.746988)
Supplement: Supplementary file 1 [file Data_Sheet_1.docx]

**Supplementary material**

**Supplementary tables**

Table S1. Multivariable logistic regression model predicting receipt of metoprolol versus observation for patients with PCI-related PMI in the unweighted study population

Table S2. Double robust analysis (IPTW with multivariate regression) of metoprolol treatment on MACEs and its components at 1-, 2-, and 3-years following PCI-related PMI

Table S3. Results of covariates in Cox regression analysis

**Supplementary figure**

Figure S1. Kernel density plots of propensity scores distribution

**Table S1.** Multivariable logistic regression model predicting receipt of metoprolol versus observation for patients with PCI-related PMI in the unweighted study population

|  | OR [95% CI] | P-value |  |
| --- | --- | --- | --- |
| **Patient characteristics** |  |  |  |
| Age | 0.974 [0.956 to 0.992] | 0.006* |  |
| Male | 0.827 [0.528 to 1.296] | 0.407 |  |
| Hypertension | 0.896 [0.568 to 1.415] | 0.639 |  |
| Current drinker | 0.621 [0.372 to 1.036] | 0.068 |  |
| Diabetes | 1.193 [0.783 to 1.819] | 0.411 |  |
| BMI | 1.040 [0.978 to 1.107] | 0.209 |  |
| Ejection fraction | 0.979 [0.962 to 0.995] | 0.011* |  |
| Length of hospitalization | 0.997 [0.956 to 1.039] | 0.874 |  |
| **Laboratory data** |  |  |  |
| Elevation of troponin I, 100% | 1.003 [0.807 to 1.245] | 0.982 |  |
| White blood cell | 1.030 [0.943 to 1.125] | 0.513 |  |
| Hemoglobin | 0.954 [0.846 to 1.076] | 0.440 |  |
| Lipoprotein (a) | 1.004 [0.997 to 1.011] | 0.305 |  |
| HDL | 2.220 [1.110 to 4.442] | 0.024* |  |
| LDL | 0.733 [0.585 to 0.919] | 0.007* |  |
| Uric acid | 0.999 [0.997 to 1.000] | 0.156 |  |
| **PCI data** |  |  |  |
| CTO | 1.740 [0.985 to 3.074] | 0.056 |  |
| LAD | 1.154 [0.763 to 1.745] | 0.498 |  |
| LM | 0.441 [0.229 to 0.850] | 0.014* |  |
| LCX | 1.532 [0.935 to 2.512] | 0.091 |  |
| Total bilirubin | 1.010 [0.984 to 1.038] | 0.448 |  |
| **Medication** |  |  |  |
| ARB | 1.152 [0.764 to 1.736] | 0.499 |  |
| CCB | 0.542 [0.358 to 0.821] | 0.004* |  |
| ACEI | 1.530 [1.025 to 2.284] | 0.037* |  |
| Trimetazidine | 0.778 [0.538 to 1.126] | 0.184 |  |
| Statin | 0.494 [0.146 to 1.670] | 0.257 |  |

Refer to Table 1 for abbreviations.

*P<0.05

**Table S2.** Double robust analysis (IPTW with multivariable regression) of metoprolol treatment on MACEs and its components at 1-, 2-, and 3-years

|  | 3 years |  |  |  | 2 years |  |  |  | 1 year |  |  |
| --- | --- | --- | --- | --- | --- | --- | --- | --- | --- | --- | --- |
|  | events/patients (%) | OR [95% CI] | *P*-value |  | events/patients (%) | OR [95% CI] | *P*-value | | events/patients (%) | OR [95% CI] | *P*-value |
| MACE | 165/860 (19.2) | 0.935 [0.877 to 0.996] | 0.038* |  | 123/860 (14.3) | 0.913 [0.862 to 0.966] | 0.002* |  | 46/860 (5.3) | 0.972 [0.943 to 1.005] | 0.080 |
| Cardiac death | 29/860 (3.4) | 1.003 [0.973 to 1.034] | 0.857 |  | 16/860 (1.9) | 0.991 [0.970 to 1.012] | 0.411 |  | 3/860 (0.3) | 1.006 [0.995 to 1.018] | 0.309 |
| Myocardial infarction | 12/860 (1.4) | 0.972 [0.945 to 0.998] | 0.037* |  | 7/860 (0.8) | 0.977 [0.954 to 1.002] | 0.063 |  | 3/860 (0.3) | 0.996 [0.983 to 1.011] | 0.588 |
| Stroke | 3/860 (0.3) | 1.008 [0.998 to 1.019] | 0.124 |  | 2/860 (0.2) | 1.001 [0.996 to 1.009] | 0.775 |  | 0/860 (0) | NA |  |
| Revascularization | 125/860 (14.5) | 0.943 [0.892 to 0.997] | 0.039* |  | 99/860 (11.5) | 0.934 [0.889 to 0.982] | 0.007* |  | 40/860 (4.7) | 0.965 [0.934 to 0.998] | 0.035* |

Double robust analysis additionally adjusted covariates with SMD ≥ 0.05 after IPTW adjustment, which were heart rate on admission, statin usage, and NT-proBNP.

Refer to Table 1 for abbreviations.

*P<0.05

**Table S3.** Results of covariates in Cox regression analysis

|  | Statin use |  |  | NT-proBNP |  |  | Heart rate |  |
| --- | --- | --- | --- | --- | --- | --- | --- | --- |
|  | HR [95% CI] | *P*-value |  | HR [95% CI] | *P*-value |  | HR [95% CI] | *P*-value |
| MACEs | 1.317 [0.182 to 9.391] | 0.797 |  | 1.002 [0.993 to 1.022] | 0.798 |  | 1.001 [0.990 to 1.012] | 0.868 |
| Cardiac death | 0.955 [0.352 to 4.199] | 0.947 |  | 0.990 [0.938 to 1.053] | 0.746 |  | 0.997 [0.986 to 1.008] | 0.606 |
| Revascularization | 0.972 [0.133 to 6.937] | 0.980 |  | 0.993 [0.952 to 1.043] | 0.776 |  | 0.999 [0.987 to 1.012] | 0.885 |
| Stroke | NA |  |  | NA |  |  | NA |  |
| MI | 1.239 [0.430 to 2.614] | 0.655 |  | 0.994 [0.950 to 1.048] | 0.822 |  | 1.003 [0.991 to 1.018] | 0.675 |

Refer to Table 1 for abbreviations.

**Figure S1.** Kernel density plots of propensity scores distribution


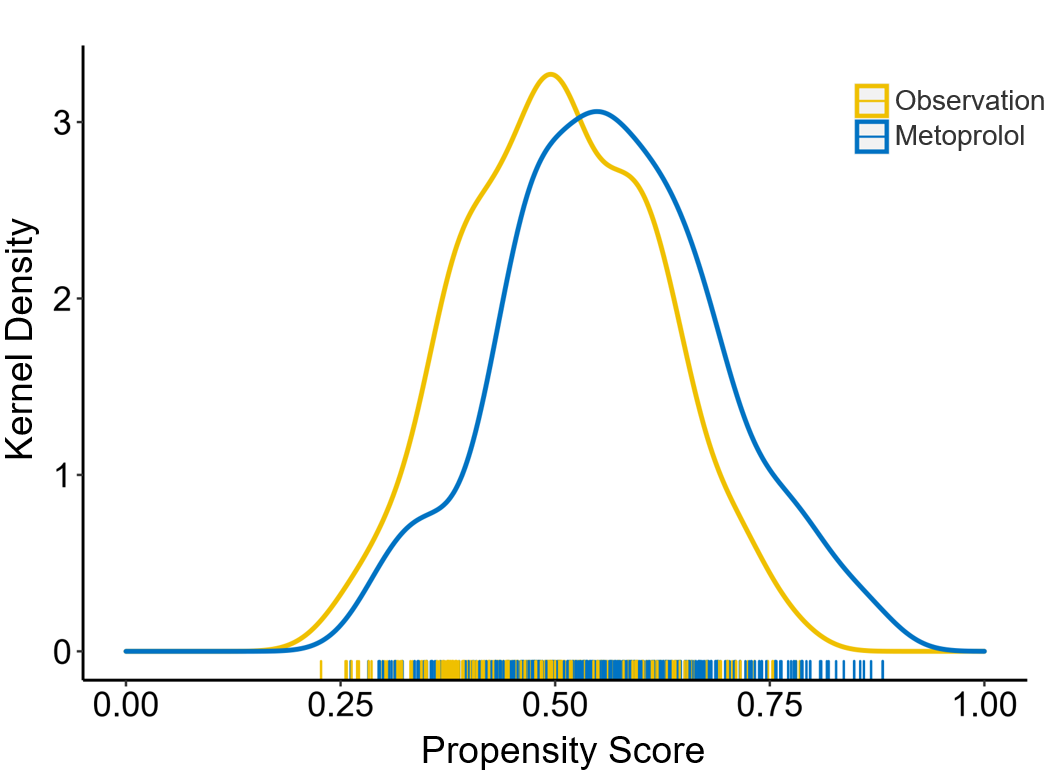


Kernel density plots show the propensity scores distribution of metoprolol and observation groups after inverse probability of treatment weighting adjustment.
